# Immunogenicity of a virosomally-formulated *Plasmodium falciparum *GLURP-MSP3 chimeric protein-based malaria vaccine candidate in comparison to adjuvanted formulations

**DOI:** 10.1186/1475-2875-10-359

**Published:** 2011-12-13

**Authors:** Marco Tamborrini, Sabine A Stoffel, Nicole Westerfeld, Mario Amacker, Michael Theisen, Rinaldo Zurbriggen, Gerd Pluschke

**Affiliations:** 1 Swiss Tropical and Public Health Institute, Socinstr. 57, CH 4002 Basel, Switzerland; 2 University of Basel, Petersplatz 1, CH 4003 Basel, Switzerland; 3 Pevion Biotech AG, Worblentalstrasse 32, CH-3063 Ittigen, Switzerland; 4 Mymetics S.A., 4 routes de la Corniche, CH-1066 Epalinges, Switzerland; 5 Department of Clinical Biochemistry and Immunology, State Serum Institute, Artillerivej 5, 2300 Copenhagen S, Denmark; 6 Lonza AG, CH-3930 Visp, Switzerland

**Keywords:** Influenza virosomes, GMZ2 immunogenicity, Vaccine candidate, *Plasmodium falciparum *malaria

## Abstract

**Background:**

In clinical trials, immunopotentiating reconstituted influenza virosomes (IRIVs) have shown great potential as a versatile antigen delivery platform for synthetic peptides derived from *Plasmodium falciparum *antigens. This study describes the immunogenicity of a virosomally-formulated recombinant fusion protein comprising domains of the two malaria vaccine candidate antigens MSP3 and GLURP.

**Methods:**

The highly purified recombinant protein GMZ2 was coupled to phosphatidylethanolamine and the conjugates incorporated into the membrane of IRIVs. The immunogenicity of this adjuvant-free virosomal formulation was compared to GMZ2 formulated with the adjuvants Montanide ISA 720 and Alum in three mouse strains with different genetic backgrounds.

**Results:**

Intramuscular injections of all three candidate vaccine formulations induced GMZ2-specific antibody responses in all mice tested. In general, the humoral immune response in outbred NMRI mice was stronger than that in inbred BALB/c and C57BL/6 mice. ELISA with the recombinant antigens demonstrated immunodominance of the GLURP component over the MSP3 component. However, compared to the Al(OH)_3_-adjuvanted formulation the two other formulations elicited in NMRI mice a larger proportion of anti-MSP3 antibodies. Analyses of the induced GMZ2-specific IgG subclass profiles showed for all three formulations a predominance of the IgG1 isotype. Immune sera against all three formulations exhibited cross-reactivity with *in vitro *cultivated blood-stage parasites. Immunofluorescence and immunoblot competition experiments showed that both components of the hybrid protein induced IgG cross-reactive with the corresponding native proteins.

**Conclusion:**

A virosomal formulation of the chimeric protein GMZ2 induced *P. falciparum *blood stage parasite cross-reactive IgG responses specific for both MSP3 and GLURP. GMZ2 thus represents a candidate component suitable for inclusion into a multi-valent virosomal malaria vaccine and influenza virosomes represent a versatile antigen delivery system suitable for adjuvant-free immunization with recombinant proteins.

## Background

The currently available strategies for malaria control rely on destruction of malaria parasites with drugs and the anopheline vectors with insecticides [1]. This combined approach proved remarkably effective in Europe and North America, whereas malaria continues to represent a huge burden in sub-Saharan Africa, Asia and South and Central America mainly due to drug-resistant parasites and insecticide resistant vectors [2]. A malaria vaccine is anticipated to be the most effective public health tool for changing this situation.

The expected outcome of modern malaria vaccine development has shifted from protecting against the parasite to surviving with the parasite, but without experiencing the noxious effects it can cause. Asexual blood-stage vaccines are aimed to primarily protect against the clinical symptoms of severe and mild malaria disease, and not against the infection, on the assumption that inhibition of parasite invasion cycles will lead to reduced parasite burden and decreased morbidity and mortality [3]. Merozoite surface proteins are, therefore, a major focus of research for blood-stage vaccines. One of the leading candidates for an anti-falciparum vaccine is GMZ2, a fusion protein consisting of the N-terminal portion of the Glutamate Rich Protein (GLURP) genetically fused to a C-terminal fragment of Merozoite Surface Protein 3 (MSP3) [4]. Data supporting MSP3 and GLURP as vaccine candidates rely on *in vitro *and *in vivo *preclinical models and on immuno-epidemiological studies demonstrating a statistically significant association between protection from clinical malaria and antigen recognition by exposed individuals [5-13].

Development of an effective vaccine against blood-stage infection will depend not only on antigen quality, but also on the choice of an optimal antigen delivery platform. In general, research on subunit vaccines focused mainly on antigen discovery whereas the method for inducing appropriate immune responses against these antigens has received less attention [14]. The selection of immunopotentiators, however, can have critical effects on safety, stability, immunogenicity and, consequently, efficacy of a vaccine [15]. The paucity of delivery systems is apparent by the fact that aluminium salts identified as immunopotentiators more than 70 years ago have remained the most common type of adjuvant licensed worldwide. Alum is regarded as safe and as a stimulator of Th2 immunity and is, therefore, used as a standard to compare to other adjuvants [16]. The need for more effective antigen delivery systems for use in vaccines against malaria is made clear by the poor responses to synthetic and recombinant malarial antigens seen with the use of Alum [17-20]. Two such antigen delivery systems are Montanide ISA 720 and immunopotentiating reconstituted influenza virosomes (IRIV). The experimental Montanide ISA 720 adjuvant forms water-in-oil droplets intended to give a slow release of antigens at the injection site [21]. Montanide ISA 720 based formulations have been shown to elicit high antibody titres in several animal species [22], and have also been investigated in malaria vaccine trials [23-26]. IRIVs are spherical, unilamellar vesicles, prepared by detergent removal from a mixture of natural and synthetic phospholipids and influenza surface glycoproteins. The haemagglutinin membrane glycoprotein of the influenza virus is a fusion-inducing component, which facilitates antigen delivery to immunocompetent cells. IRIVs represent a universal antigen delivery system for multicomponent subunit vaccines, as antigens can be either coupled to their surface to elicit CD4 T cell and antibody responses or encapsulated in their lumen to elicit CD8 T cell responses. Experience with two licensed vaccines based on virosomes (Inflexal^® ^V and Epaxal^®^) as well as clinical trials with virosomally-formulated malaria vaccine candidates [27,28] have shown that IRIV-based formulations are immunogenic even in children and infants, and display a very good safety profile [29,30].

In the present report, a hybrid MSP3/GLURP recombinant protein (named GMZ2) was coupled to the surface of IRIVs. The immunogenicity of this formulation was compared to vaccine candidate preparations of GMZ2 in Montanide ISA 720 and Alum in three different mouse strains.

## Methods

### GMZ2 hybrid recombinant protein production and candidate vaccine formulations

Production and purification of the recombinant GMZ2 hybrid molecule, a fusion protein containing the regions GLURP_27-500 _(GLURP-R0) and MSP3_212-380 _(MSP3 C-terminal), has been described in detail elsewhere [4]. To prepare GMZ2-IRIV, 5 nmol of the purified hybrid protein was activated by incubation with 100 nmol of sulfosuccinimidyl 4-[N-maleimidomethyl] cyclohexane-1-carboxylate (Sulfo-SMCC; Thermo Scientific) for 1 h at room temperature. This was followed by conjugation to 1, 2-dipalmitoyl-sn-glycero-3-phosphothioethanol (Avanti Polar Lipids) for 4 h at room temperature, prior to incorporation into influenza virosomes, as described in [31]. Virosomes were aliquoted and stored at 4°C. The mixture of GMZ2 with Alum (Al(OH)_3_) was prepared as follows: 0.23 mg of GMZ2 protein was diluted in 3.7 mL of PBS pH 7.4, and 0.93 mL of Alum suspension (Imject Alum, 10 mg/mL; Thermo Scientific) was added dropwise under continuous mixing. The solution was aliquoted and stored at 4°C. The mixture of GMZ2 with Montanide was prepared as follows: 0.05 mg of GMZ2 protein was diluted in 0.38 mL of PBS pH 7.4, and 0.5 mL of Montanide ISA 720 solution (Seppic SA, France) was added dropwise under continuous mixing. This was followed by vigorous pushing through a syringe needle (18 G 1.5", 1.2 × 40 mm) for 25 times. This solution was used within 24 h after preparation.

### Mouse immunogenicity studies

**Figure 1:**
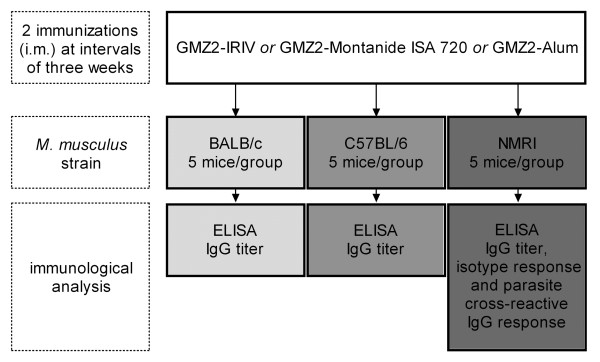
**GMZ2 formulations, immunization protocol and analysis schedule**.

BALB/c, C57BL/6, and NMRI mice (five animals/group) purchased by Charles River were pre-immunized intramuscularly with 0.1 mL of inactivated influenza virus (Influenza A/Singapore/6/86 (H1N1), 1 μg of haemagglutinin; Berna Biotech, Switzerland). Induction of influenza antigen-specific CD4 T-cells can provide T-cell help for B cells that recognize the foreign malaria antigen on the surface of IRIVs and thus enhance the immune response to the GMZ2 protein. Three- and six weeks later, mice were immunized with a dose of 5 μg GMZ2 administered either in association with Alum, Montanide ISA 720, or IRIVs (Figure 1). Approval for animal experimentation has been obtained from the responsible authorities and all animal manipulations have been performed under controlled laboratory conditions by specifically qualified personnel in strict accordance with the Rules and Regulations for the Protection of Animal Rights laid down by the Swiss Bundesamt für Veterinärwesen.

### ELISA

ELISA MaxiSorp microtitre plates (Nunc, Dr. Grogg, Stetten-Deiswill, Switzerland) were coated at 4°C overnight with 1 μg/mL GMZ2 in carbonate buffer (0.05 M; pH 9.4) or with the individual GLURP and MSP3 recombinant proteins. Production and purification of the GLURP and MSP3 proteins has been described elsewhere [4]. Wells were then blocked with 5% milk powder in PBS pH 7.4 for 1 h at 37°C followed by three washings with PBS containing 0.05% Tween-20. Plates were incubated with two-fold serial dilutions of sera starting with 1:50 in PBS pH 7.4 containing 0.05% Tween-20 and 0.5% milk powder for 2 h at 37°C. After washing, the plates were incubated with horseradish-peroxidase conjugated goat anti-mouse IgG antibodies (BD; 1:2000 in PBS containing 0.05% Tween-20 and 0.5% milk powder) for 1 h at 37°C and then washed. One OPD tablet (Sigma, Switzerland) was dissolved in citrate-buffer and 0.04% H_2_O_2 _(v/v) was added followed by an incubation at room temperature in the dark. After 5 min the reaction was stopped by addition of sulphoric acid (Merck, Darmstadt, Germany) to reach a final concentration of 0.5 M. The optical density (OD) of the reaction product was recorded at 492 nm using a microplate reader (SpectraMax plus, Bucher Biotech, Basel, Switzerland). Titration curves were registered using Softmax PRO software. Endpoint titres were calculated by comparing the ELISA OD of the test serum with the ELISA OD of PBS. Endpoint titre is the last serum dilution where the OD of the test sera > 2 × OD_492 _of the negative control (PBS).

The anti-GMZ2, GLURP and MSP3 IgG subclasses were determined by ELISA as described above. HRP-conjugated goat anti-mouse IgG1, IgG2a, IgG2b and IgG3 (Caltag, UK) were used at a 1:2000 dilution in PBS. The results are expressed as mean OD_492 _+ S.D.

### Indirect immunofluorescence assay (IFA)

*Plasmodium falciparum *strain 3D7 was cultured essentially as described previously [32]. Synchronized *P. falciparum *schizonts were washed and mixed with two volumes of a solution containing 4% (v/v) paraformaldehyde and 0.1% (v/v) Triton X-100. Droplets of 40 μL of cell suspension were added to each well of a diagnostic microscope slide (Flow Laboratories, Baar, Switzerland) and incubated for 30 min at room temperature. Cells were blocked with blocking solution containing 100 mg/mL fatty acid-free bovine serum albumin in PBS pH 7.4. Immunostaining was performed by incubating the wells with 25 μL of an appropriate serum dilution in blocking solution in a humid chamber for 1 h at room temperature. In competition experiments primary antibodies were pre-incubated for 30 min with recombinant competitors at a concentration of 10 μg/mL. After five washes with blocking solution, 25 μL of 5 μg/mL indocarbocyanine dye-conjugated affinity-pure F(ab')_2 _fragment goat anti-mouse IgG heavy-chain antibodies (Jackson ImmunoResearch Laboratories, West Grove, Pa.), diluted in blocking solution were added to the wells and incubated for 1 h at room temperature. Finally, the wells were washed five times, mounted with ProLong^® ^Gold antifade reagent with DAPI (Invitrogen) and covered with a coverslip. Antibody binding and DNA staining were assessed by fluorescence microscopy.

### Sodium dodecyl sulfate-polyacrylamide gel electrophoresis (SDS-PAGE) and immunoblotting

Parasite lysates were prepared by saponin lysis of *P. falciparum *3D7-infected erythrocytes. Cultured parasites were collected at the schizont stage and washed three times with RPMI medium. Pelleted infected red blood cells were lysed by mixing with a large volume (adjusted to 5% haematocrit) of 0.015% (w/v) saponin in PBS and incubated on ice for 20 min. Finally, the pelleted parasites were resuspended in PBS and stored at -80°C until further use.

A total of 50 μl of parasite lysate was solubilized in an equal volume of 2× loading buffer (1.7 mL of 0.5 M Tris-HCl [pH 6.8], 2 mL of glycerol, 4.5 mL of 10% [w/v] sodium dodecyl sulfate, 1 ml of β-mercaptoethanol, 0.8 ml of 0.3%, w/v bromophenol blue) and heated to 95°C for 10 min. Proteins were separated on an SDS-PAGE minigel and electrophoretically transferred to a nitrocellulose filter by semidry blotting. Blots were blocked with PBS containing 5% milk powder and 0.1% Tween 20 overnight at 4°C. The filter was cut into strips and incubated with appropriate dilutions of immune serum in blocking buffer for 2 h at room temperature. In competition experiments primary antibodies were pre-incubated for 30 min with recombinant competitors at a concentration of 1 μg/mL. After several washing steps, filter strips were incubated with goat anti-mouse IgG horseradish peroxidase conjugated Ig (0.1 μg/mL; Bio-Rad Laboratories, Hercules, Calif.) for 1 h. Blots were developed using the ECL (Pierce) system according to manufacturer's instructions.

## Results

### Development of anti-GMZ2 IgG responses in mice

**Figure 2:**
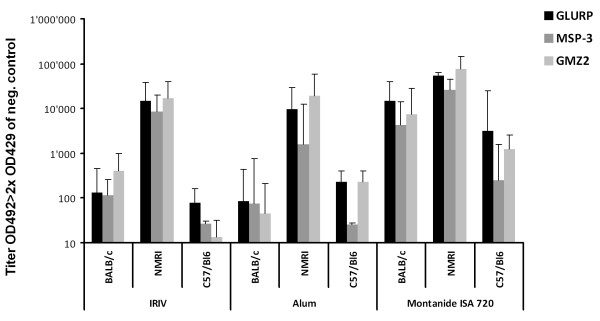
**Development of anti-GMZ2, anti-GLURP and anti-MSP3 total IgG responses in three mouse strains upon immunization with differentially-formulated GMZ2**. Shown are ELISA mean endpoint titres (+ S. D.) of individual immunization groups.

In order to examine whether an adjuvant-free IRIV formulation of the recombinant hybrid protein GMZ2 elicits *P. falciparum *cross-reactive antibody responses, highly purified GMZ2 was chemically coupled to phosphatidylethanolamine and then attached to the surface of IRIVs. Since a vaccine needs to be immunogenic in genetically diverse populations, outbred and inbred mice were immunized to investigate antibody responses to GMZ2 and to evaluate the effects of immunogenetic differences. Outbred (NMRI) and inbred (BALB/c and C57BL/6) mice were immunized two times with a dose of 5 μg of GMZ2 either coupled to IRIVs or as adjuvanted formulation in combination with either Al(OH)_3 _or Montanide ISA 720. Sera collected 3 weeks after the second immunization were assessed for IgG antibody titres specific for GMZ2 and its individual GLURP and MSP3 components by ELISA (Figure 2). Significant differences in IgG antibody responses were found between the mouse strains. In outbred NMRI mice, non-adjuvanted GMZ2 coupled to IRIVs elicited comparable anti-GMZ2-specific IgG responses as the adjuvanted formulations (Figure 2). In the inbred mouse strains (BALB/c and C57BL/6), GMZ2 adjuvanted with Montanide ISA 720 was generally more immunogenic than GMZ2 on IRIVs or in Al(OH)_3_. In all immunization groups both anti-MSP3 and anti-GLURP IgG were elicited by GMZ2 immunization. In general the anti-GLURP ELISA titres were higher than anti-MSP3 titres. Compared to the Al(OH)_3_-adjuvanted formulation the two other formulations showed increased anti-MSP3-specifc titres in NMRI mice. No GMZ2-specific IgG responses were found in pre-immune sera.

**Figure 3:**
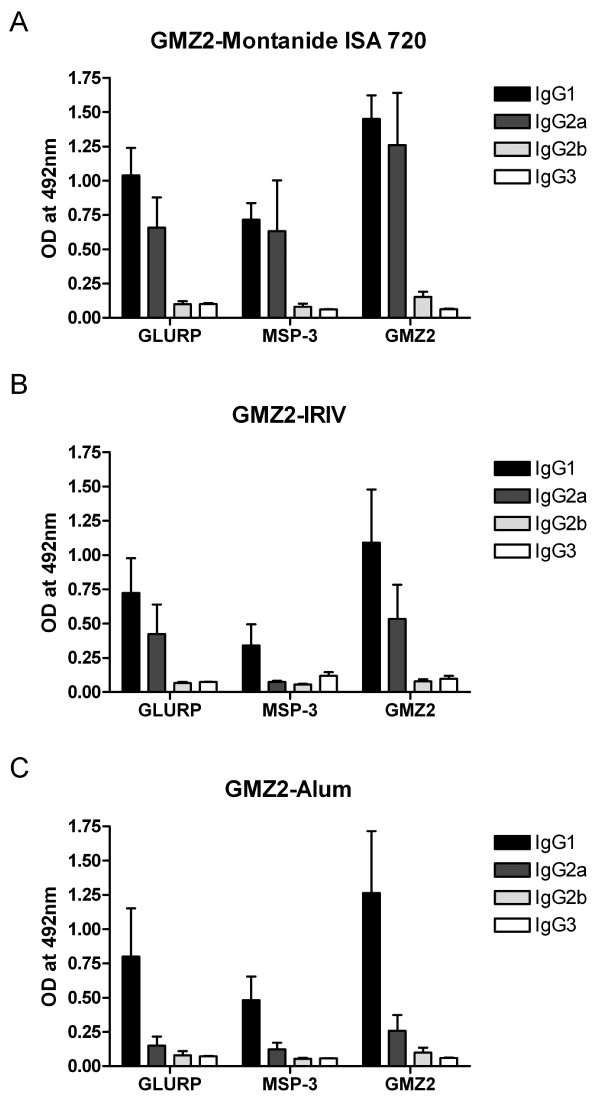
**Determination of the IgG subclass profiles by ELISA using GMZ2 and the individual proteins MSP3 and GLURP**. Results are expressed as mean OD492 + S.D. Sera from five NMRI mice in each group collected after the third immunization were tested individually. Shown are results of a single dilution (1:6400 for sera of mice immunized with Montanide ISA 702 adjuvanted GMZ2 (**a**), 1:800 for sera of mice immunized either with IRIV (**b**) or Alum (**c**) formulated GMZ2.

Since outbred mice represent better the genetic variability of humans and showed the highest ELISA titres of all three formulations, GMZ2-specific IgG subclass levels were only assessed in NMRI mice (Figure 3). All three formulations elicited predominantly IgG1. While the Montanide ISA 720 formulation elicited significant anti-GMZ2, MSP3 and GLURP IgG2a levels, IgG2a levels were comparatively low for the Alum preparation. GMZ2 coupled to IRIVs also induced substantial anti-GMZ2 and GLURP IgG2a levels. Anti-GMZ2 IgG2b and IgG3 levels were low for all three formulations.

### Induction of parasite cross-reactive IgG antibodies

**Figure 4:**
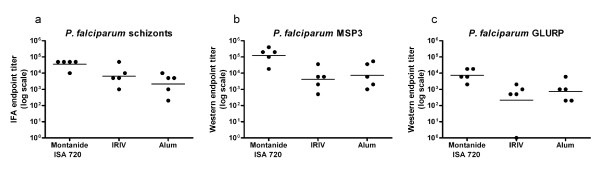
**Parasite cross-reactivity of anti-GMZ2 IgG responses**. Immune sera of differentially-formulated NMRI mice were tested for parasite binding in IFA (**a**) with *in vitro *cultured *P. falciparum *blood stage parasites and Western blot analysis (**b**, **c**) with parasite lysate. Shown are endpoint titres observed with sera of individual animals, the horizontal lines are representing the geometric mean.

**Figure 5:**
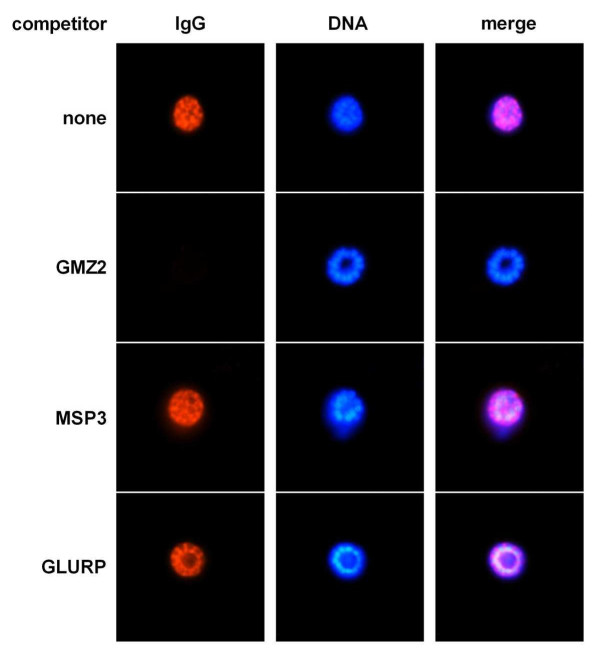
**Competition immunofluorescence staining of *in vitro *cultivated *P. falciparum *schizonts with recombinant GMZ2, MSP3 or GLURP**. Parasites were stained with anti-GMZ2 immune serum of a NMRI mouse pre-incubated without or with recombinant competitors. Immune serum was used at a dilution of 1:1000 and competitors at a concentration of 10 μg/mL. Nuclei were visualized using DAPI staining.

**Figure 6:**
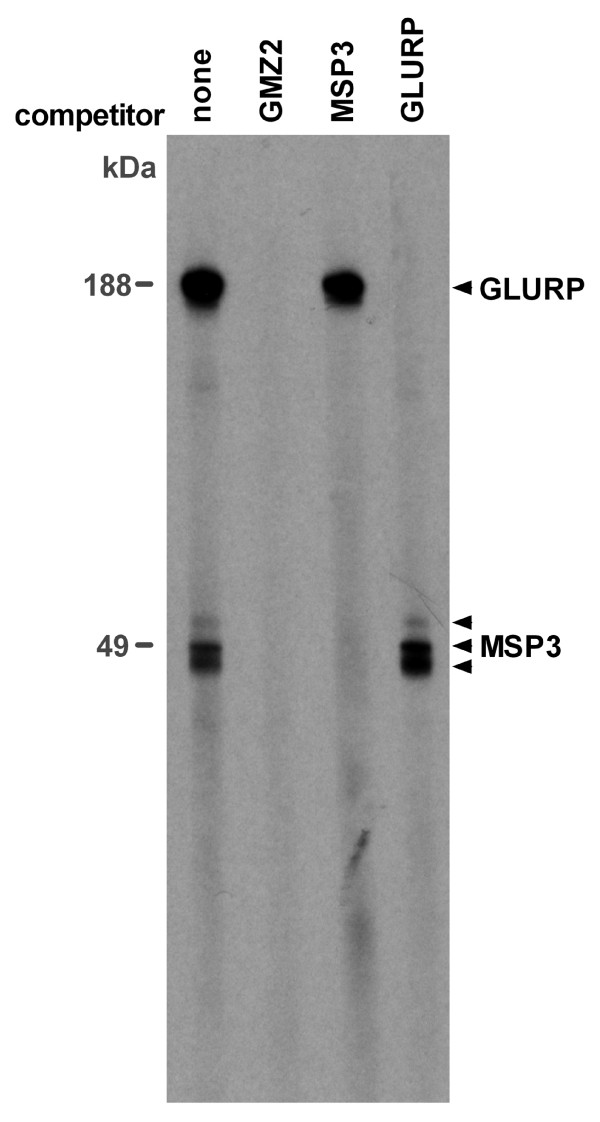
**Competition Western blot analysis with lysates from *P. falciparum *blood stage parasites and recombinant GMZ2, MSP3 or GLURP**. Total lysates of *in vitro *cultivated parasites were separated by SDS-PAGE under reducing conditions and blotted onto a nitrocellulose membrane. Serum of a NMRI mouse immunized with IRIV-formulated GMZ2 was pre-incubated with or without recombinant competitor protein and subsequently added to cut strips. Immune serum was used at a dilution of 1:2000 and competitors at a concentration of 1 μg/mL.

Induction of *P. falciparum *blood stage cross-reactive IgG upon immunization with GMZ2 was analyzed by immunofluorescence analysis (IFA) and immunoblotting with sera from the immunized NMRI mice, since these mice showed the highest ELISA titres of all three formulations. All animals developed a parasite cross-reactive IgG response. The Montanide ISA 720 based formulation elicited generally the highest mean IFA (Figure 4a) and Western blot (Figure 4b, c) titres. In contrast to the ELISA results, in Western blot analyses with parasite lysates the GLURP immunoblot IgG levels were lower than MSP3 specific IgG titres for all formulations (Figure 4b, c). However, this discrepancy could be related to different expression levels of the two individual malaria proteins. In competition IFA, staining of blood stage parasites by anti-GMZ2 antiserum was completely blocked by recombinant GMZ2, but not with the individual GLURP or MSP3 recombinant proteins (Figure 5). Residual GLURP and MSP3 specific IFA staining patterns were similar. Western blot competition experiments allowed to assess the MSP3 and GLURP cross-reactive antibody responses. *P. falciparum*-derived proteins were stained with anti-GMZ2 mouse antiserum pre-incubated with or without the recombinant competitor proteins GMZ2, MSP3 or GLURP (Figure 6). Competition with recombinant MSP3 did not abolish staining of the high molecular weight parasite-derived GLURP [4]. Competition with recombinant GLURP left a triple band unchanged, which is characteristic for processed MSP3 [33]. None of the pre-immune sera was positive in IFA or Western blot.

## Discussion

The development of protein subunit vaccines is often hampered by limited intrinsic immunostimulatory properties and the lack of cross-reactivity of elicited antibodies with the native target antigens. In an attempt to overcome both problems, the immunogenicity of a non adjuvanted virosomal formulation of the GMZ2 recombinant antigen was systematically evaluated in comparison to GMZ2 adjuvanted in Al(OH)_3 _and Montanide ISA 720 in outbred and inbred mice. The GMZ2 candidate malaria vaccine antigen is a chimeric protein consisting of the conserved N-terminal portion of GLURP genetically fused to the conserved C-terminal portion of MSP3 [4]. All GMZ2 vaccine formulations induced antibody responses in all mouse strains tested. IgG titres were higher in outbred NMRI mice than in the two inbred mice strains. Previously tested non-adjuvanted GMZ2 formulations have elicited either no or only very weak antibody responses [34]. However, in the present study anti-GMZ2-specific IgG responses elicited by the non-adjuvanted GMZ2 coupled to IRIVs were comparable to those found for adjuvanted formulations. Compared to the Alum adjuvanted formulation the virosomal preparation increased the proportion of MSP3-specifc IgG antibodies, thereby balancing the immunogenicity between the two individual domains. This finding is potentially important as previous studies in humans [35], Saimiri monkeys [36] and mice [4] have consistently demonstrated that the GLURP domain of GMZ2 is more immunogenic than the MSP3 domain. The reason for this increase in immunogenicity of the MSP3 domain is not known, but it may be speculated that the display of GMZ2 on the surface of the virosomes increases the accessibility of B-cell epitopes in MSP3.

Several parameters may influence IgG subclass profiles of antibody responses towards proteins, including antigen dosage, use of adjuvants or delivery platforms, and the intrinsic immunogenicity of the protein itself [37]. All GMZ2 vaccine formulations tested here induced predominantly subclass IgG1 responses in mice. It is well established that the addition of immune modulators can influence the sub-class profile and it was recently demonstrated that the addition of the TLR4 agonist GLA to oil-in-water or Al(OH)_3 _formulations of GMZ2 effectively leads to an increase in vaccine-specific cytophilic IgG2a and IgG2b antibodies and in particular an increase in anti-MSP3-specific titres in CB6F1 mice [34]. Thus, it may be speculated that the addition of TLR agonists like GLA to the GMZ2 virosomal formulation could enhance the overall IgG responses against GMZ2 and modulate the IgG sub-class profile in mice. Although, the use of potent adjuvants can increase immunogenicity they can also be associated with increased reactogenicity. In human clinical trials, injections of the water-in-oil emulsion Montanide ISA 720 alone were well tolerated [38]. However, some vaccine formulations with Montanide ISA 720 were quite reactogenic [25,39-41] and issues regarding antigen stability in long-term formulations were also reported [42]. Recently conducted safety and immunogenicity clinical trials with aluminium hydroxide adjuvanted GMZ2 have shown that the vaccine candidate is well tolerated, safe and immunogenic in malaria-exposed and non-exposed healthy adults [35,43] and young children [44]. Phase 2 efficacy trials are ongoing. It would be expected that a non-adjuvanted virosomal formulation would be equally safe since the influenza virosome antigen delivery platform has already been commercialized and over 70 million doses of virosome-based vaccines have been administered in humans over the last 10 years, including children and infants [29,30,45]. Moreover, the virosome antigen delivery platform has already proven its suitability for malaria vaccine design exhibiting an excellent safety profile. Virosomes formulated with *P. falciparum *peptides from both the pre-erythrocytic antigen CSP and the blood-stage antigen AMA-1, have been successfully tested in clinical phase Ia and IIa trials [27,28]. A phase Ib trial using this two-peptide formulation has recently been finalized [45]. As an exploratory outcome, the incidence rate of clinical malaria episodes in children vaccinees was half the rate of the control children. These promising results support the concept to develop a multivalent virosomal malaria vaccine by incorporating additional components, such as GMZ2, into the clinically tested bivalent formulation.

## Conclusions

Mouse immunogenicity data presented here, demonstrate that a non-adjuvanted virosomal formulation of the chimeric GMZ2 protein elicits high titres of parasite cross-reactive antibodies. These results confirm that virosomes represent a versatile antigen delivery platform for multi-valent vaccines based on recombinant proteins, synthetic peptides, and/or carbohydrates.

## Competing interests

Sabine A Stoffel and Mario Amacker are employees of Pevion Biotech AG.

## Authors' contributions

MiT, RZ and GP designed the research. MaT, SAS, NW and MA performed the research. MaT, SAS, MiT, and GP analysed the data. MaT and GP wrote the paper with contributions from the other authors.

All authors read and approved the final manuscript.
